# P-Channel InGaN/GaN heterostructure metal-oxide-semiconductor field effect transistor based on polarization-induced two-dimensional hole gas

**DOI:** 10.1038/srep23683

**Published:** 2016-03-29

**Authors:** Kexiong Zhang, Masatomo Sumiya, Meiyong Liao, Yasuo Koide, Liwen Sang

**Affiliations:** 1 International Center for Materials Nanoarchitectonics (MANA), National Institute for Materials Science (NIMS), 1-1 Namiki, Tsukuba, Ibaraki 305-0044, Japan; 2 Wide Bandgap Materials Group, National Institute for Materials Science (NIMS), 1-1 Namiki, Tsukuba, Ibaraki 305-0044, Japan; 3 JST-PRESTO, The Japan Science and Technology Agency, Tokyo 102-0076, Japan

## Abstract

The concept of *p*-channel InGaN/GaN heterostructure field effect transistor (FET) using a two-dimensional hole gas (2DHG) induced by polarization effect is demonstrated. The existence of 2DHG near the lower interface of InGaN/GaN heterostructure is verified by theoretical simulation and capacitance-voltage profiling. The metal-oxide-semiconductor FET (MOSFET) with Al_2_O_3_ gate dielectric shows a drain-source current density of 0.51 mA/mm at the gate voltage of −2 V and drain bias of −15 V, an ON/OFF ratio of two orders of magnitude and effective hole mobility of 10 cm^2^/Vs at room temperature. The normal operation of MOSFET without freeze-out at 8 K further proves that the *p*-channel behavior is originated from the polarization-induced 2DHG.

The conventional Si-based complementary logic integrated circuits (ICs) show the drawbacks of large leakage current and poor reliability in harsh environments^1^. Wide-bandgap semiconductor III-Nitrides provide a better choice for the logic ICs applications owing to their superior physical and chemical properties, such as the high breakdown voltage, high thermal stability, large saturation velocity, and high carrier mobility at heterojunctions^2^,^3^. To achieve the complementary ICs, both *n*- and *p*-channel field effect transistors (FETs) with well-matched performance are necessary^4^. Recently, III-Nitrides *n*-channel heterojunction FETs using polarization-induced two-dimensional electron gas (2DEG) have already been extensively investigated with lower specific on-resistance and higher electron mobility compared with those of Si-based FETs^5^. On the other hand, little attention has been paid to *p*-channel FETs because of the difficulty in obtaining hole with high charge density and high mobility. The unbalanced development between *n*-channel and *p*-channel FETs makes the complementary ICs using III-Nitrides a great challenge, implying that the research on III-Nitrides *p*-channel FETs is in great demand.

According to the polarization theory, two-dimensional hole gas (2DHG) with high charge density and high mobility can be induced by negative polarization charge at the III-Nitrides heterointerface similarly to that for 2DEG^6^,^7^,^8^,^9^,^10^,^11^. The 2DHG and related *p*-channel FETs have been proposed in Al(Ga)N- and AlInGaN-based heterostructure^12^,^13^,^14^,^15^,^16^. Compared to the AlGaN system, InGaN offers a better route for the *p*-channel transistors in term of a much lower on-resistance because of its lower activation energy of Mg dopants and hole effective mass^17^,^18^. In addition, the adjustable compressive strain in InGaN increases the deformation of valence bands, and splits the degeneracy between light hole and heavy hole bands^19^, which also enhances the hole mobility^20^. Moreover, InGaN presents higher piezoelectric polarization coefficient compared to its III-nitrides rivals, which is beneficial for the generation and accumulation of high density 2DHG in conducting channel^21^. It has been theoretically simulated that for the compressively strained InGaN layer on the relaxed GaN template, holes can be confined close to the lower interface as a result of the dominant piezoelectric polarization field^22^,^23^,^24^,^25^,^26^. However, it is still difficult to experimentally extract high-density 2DHG in the InGaN system. Therefore, although *p*-channel metal-semiconductor FET (MESFET) has been reported by using InGaN/GaN heterostructure, its performances such as leakage current, drain-source current density (0.01 mA/mm), ON/OFF ratio remain still quite poor^25^.

In order to improve the performance of InGaN-based *p*-channel FETs, the structural optimization for an effective 2DHG and novel device concepts are in great demand. In this paper, a super-thin ultra-flat GaN spacer layer is proposed between InGaN and high-resistance GaN template to reduce the interface roughness scattering for the 2DHG. With the optimized structure, a metal-oxide-semiconductor FET (MOSFET) using Al_2_O_3_ as gate dielectric is demonstrated for the first time. The accumulation of 2DHG with high concentration at the lower interface of InGaN/GaN is confirmed from both theoretical simulation and capacitance-voltage (*C-V*) measurement. The MOSFET shows a high drain-source current *I*_*DS*_ of 0.51 mA/mm at the gate voltage *V*_*GS*_ of −2 V and drain bias *V*_*DS*_ of −15 V, and ON/OFF ratio of two orders of magnitude at room temperature. The normal operation of MOSFET at 8 K further proves that the *p*-channel behavior is originated from the polarization-induced 2DHG.

## Results

**Figure 1:**
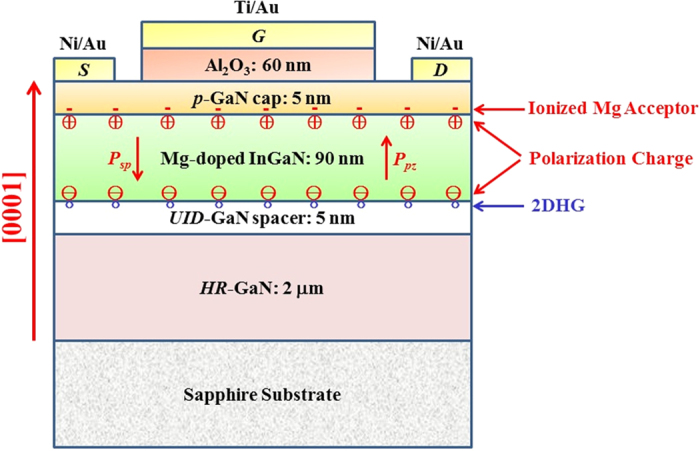
Cross-sectional schematic structure of the designed InGaN/GaN heterostructure MOSFET (not to scale) (*P*_*sp*_: spontaneous polarization, *P*_*pz*_: piezoelectric polarization).

**Figure 2:**
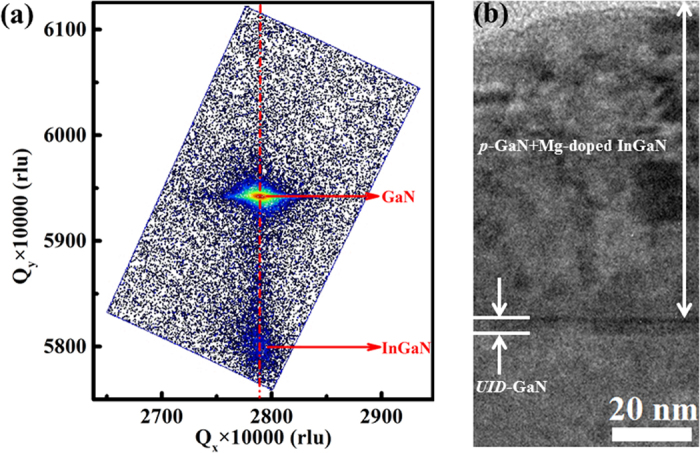
(**a**) The (10–14)-plane HRXRD reciprocal space mapping and (**b**) Bright-field cross-sectional TEM image with *g* vector of 0002 along the zone axis of [1–100] of the InGaN/GaN heterostructure.

Firstly, we designed the structure of the InGaN/GaN heterojunction by a simulation using self-consistent solution of Poisson-Schrödinger equations combined with polarization-induced theory. The band diagram, hole concentration, and distribution can be obtained from the simulations. The details of material parameters adopted during the simulations can be found elsewhere^27^. As a result of piezoelectric polarization between InGaN and GaN, high-density negative polarization charges are created at the lower interface of pseudomorphic InGaN/GaN heterostructure. To compensate these fixed charges, hole accumulation with large band bending happens near the interface of InGaN/GaN heterostructure. From the simulation, the optimized thickness for the strained InGaN is 90 nm and In composition of 25%. The optimized structure was grown by using the metal organic chemical vapor deposition (MOCVD) (see the Methods section for details). The structure in this study is schematically shown in Fig. 1. It is noted that before InGaN deposition, a long-time growth interrupt in both nitrogen and ammonia ambient was introduced to polish the interface, followed by a super-thin unintentionally doped GaN (*UID*-GaN) spacer layer with an ultra-flat morphology. The growth interrupt can improve the interface quality, which was confirmed in our previous study^28^,^29^. Slightly Mg-doping is performed for the strained InGaN layer to compensate the *n*-type background concentration^26^. Figure 2 presents the structural properties of the deposited InGaN/GaN heterojunction on GaN template. The high-resolution X-ray diffraction (HRXRD) reciprocal space mapping (RSM) around (10–14)- plane reveals that the InGaN layer is totally strained on the GaN template, ensuring a good quality with large piezoelectric polarization field (Fig. 2(a)). Figure 2(b) shows the cross-sectional bright field transmission electron microscopy (TEM) image of the InGaN/GaN heterojunction. An abrupt interface can be clearly seen at the view in a high magnification. The thickness of InGaN is about 90 nm, which is consistent with the designed structure. The black and silver dots in InGaN layer are from the focused ion beam (FIB) induced damages during the preparation of TEM specimen.

**Figure 3:**
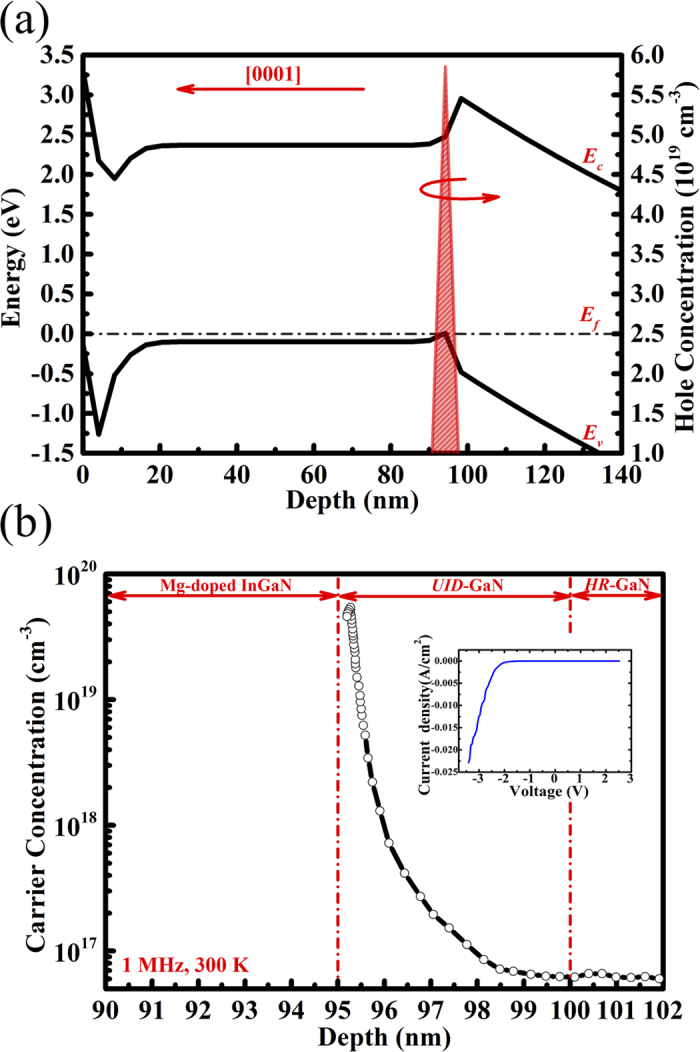
(**a**) Simulated band diagram, hole concentration and distribution of the designed InGaN/GaN heterostructure at 300 K. (**b**) Calculated hole concentration profile based on the *C-V* characteristic of the InGaN/GaN heterostructure using the Schottky contact. Inset: *I-V* characteristic of the Schottky contact.

It was simulated that the peak hole density at the InGaN/GaN heterojunction was over 5.5 × 10^19^ cm^−3^ with full width at half maximum (FWHM) value of about 2 nm, indicating the formation of the 2DHG conducting channel with a high density (Fig. 3(a)). The *C-V* characteristic for a Schottky contact with Ti/Au (40/110 nm) stacks was measured at a frequency of 1 MHz. The carrier profile dependent on the thickness extracted from *C-V* curve at 300 K indicates a hole accumulated at 95 nm from the surface, which is around the InGaN/GaN heterointerface (Fig. 3(b)). The peak density of hole is calculated to be above 5 × 10^19^ cm^−3^. It is also noted that, the Schottky behavior shows an obvious *p*-type characteristic as shown in the inset of Fig. 3(b), in which, the rectify ratio is more than 3 orders of magnitude at 3 V.

**Figure 4:**
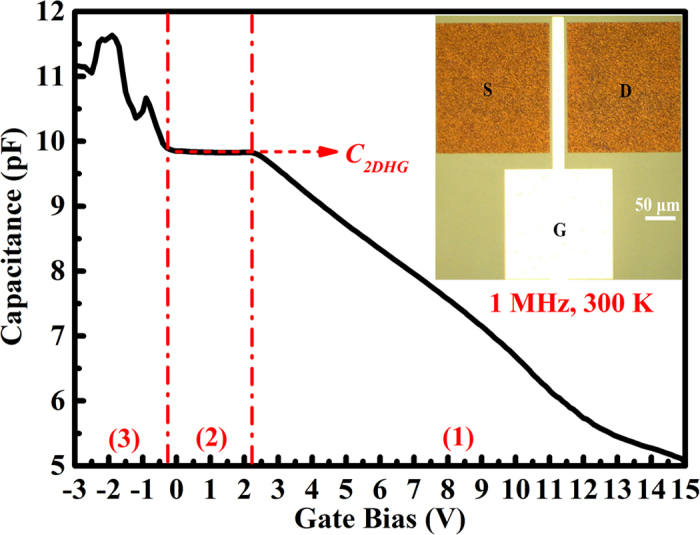
*C-V* curve of the InGaN/GaN heterostructure MOSFET at 300 K under dark condition with scan frequency of 1 MHz. Inset: microscopic image of a fabricated MOSFET device.

The fabricated InGaN/GaN heterojunction MOSFET shows an obvious 2DHG behavior. Figure 4 is the *C-V* characteristics of MOSFET at 300 K under dark condition. The high scan frequency of 1 MHz ensures that all the interface states cannot respond to the AC signal but only follows the DC gate bias. The gate bias was swept from +15 to −3 V with a step of 0.1 V. The *C-V* curve presents a two-step capacitance, which is the characteristic feature of the MOSFET structure having two effective interfaces^30^. For bias scanning from 15 to about 2 V, the measured capacitance starts to increase until reaching a plateau, which is the behavior of the depletion of the bulk. From about 2 to −0.2 V, the nearly flat capacitance *C*_*2DHG*_ indicates the formation of 2DHG accumulated at the InGaN/GaN interface. Therefore, the depletion layer changes the measured capacitance only slightly in this plateau region. At the bias below −0.2 V, holes start to distribute in the Mg-doped InGaN and *p*-GaN cap layer, which results in a slight increase of the total capacitance. With further increase of the negative bias, the Al_2_O_3_ dielectric layer starts to deplete. Because the maximum gate bias was limited to −3 V by the gate leakage current, the *C*_*Al2O3*_ corresponding to the 60 nm-thick Al_2_O_3_ was not shown here.

**Figure 5:**
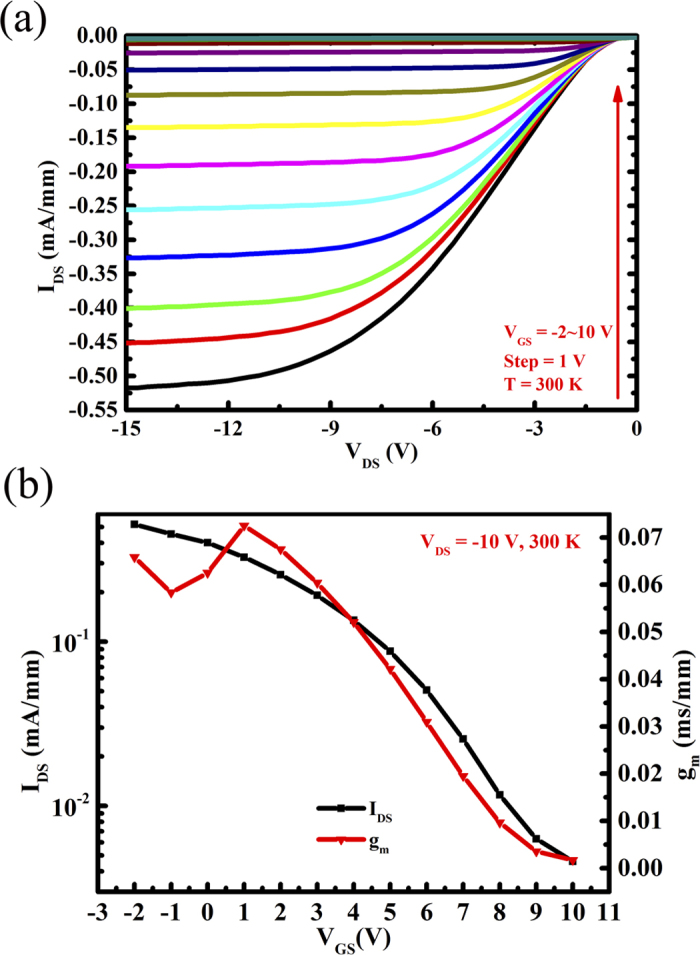
(**a**) DC output and (**b**) Semi-log plot of transfer characteristics and transconductance of the InGaN/GaN heterostructure MOSFET at 300 K.

**Figure 6:**
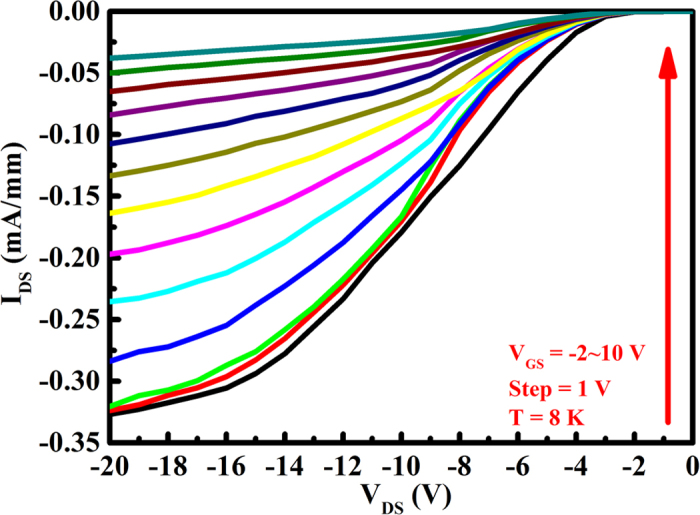
DC output characteristics of the InGaN/GaN heterostructure MOSFET at 8 K.

The DC output characteristics of the MOSFET at 300 K are displayed in Fig. 5(a). The gate-source voltage *V*_*GS*_ was varied from −2 to 10 V in steps of 1 V. The depletion-mode (D-mode) behavior with an absolute source-drain current density, /I_DS_/, 0.51 mA/mm for a negative gate voltage of −2 V is observed, which is over 40 times higher than that reported in InGaN/GaN *p*-channel HFET^25^. With increasing the positive gate voltage, the transistor presents an off behavior. An ON/OFF ratio close to two orders of magnitude is obtained for this D-mode transistor. Figure 5(b) presents the transfer characteristics and transconductance of the MOSFET at 300 K. The threshold voltage V_TH_ of the MOSFET was estimated by extrapolating the linear region down the voltage axis to be about 10 V. The maximum transconductance *g*_*m*_ is about 0.07 mS/mm at the drain bias V_DS_ of −15 V. Further improvement can be obtained by reducing the parasitic resistances, such as reducing the ohmic contact resistance, or the gate-channel separation and the device dimensions. The output characteristic of this *p*-channel MOSFET is also checked at low temperatures. Generally, if the *p*-channel performances are related to acceptor doping, a channel carrier free-out will occur at low temperatures. Since the thermal ionization energy of Mg in GaN and In_0.25_Ga_0.75_N is 160 and 54 meV, respectively, the temperature for the failure of device should be higher than 40 K^31^. However, this significant *p*-channel behavior can be still observed in our developed InGaN-based MOSFET (Fig. 6) measured as low as 8 K. The source-drain current density *I*_*DS*_is still as high as 0.32 mA/mm at the gate voltage *V*_*GS*_ of −2 V and drain bias *V*_*DS*_ of −20 V. Therefore, it can be verified that the *p*-channel characteristic is originated from not the acceptor doping but the polarization- induced 2DHG at the lower interface of InGaN/GaN heterojunction. Due to the degradation of ohmic contact and inferior performance of gate metal electrode at cryogenic temperatures, the output characteristic shows a Schottky-like behavior and could not be completely pinched off at the gate voltage of 10 V.

## Discussion

The effective mobility (*μ*_*eff*_) of the 2DHG in the channel can be extracted by using the following equation^32^:


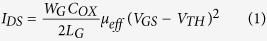


where *C*_*OX*_ is the gate oxide capacitance. The *μ*_*eff*_ values at 300/8 K are calculated to be ~10/12 cm^2^/Vs. The lower 2DHG mobility might be due to the impurity scattering, dislocation scattering, interface roughness scattering or alloy disorder scattering around the InGaN/GaN interface^16^. Moreover, due to the immature device processing of *p*-channel MOSFET, especially the unsatisfactory gate dielectric processing, the interface charges^33^, fixed charges^34^ and polarization charges^35^,^36^ along the Al_2_O_3_/*p*-GaN interface could also influence the accumulation and transportation of 2DHG in conducting channel. For the further improvement of the 2DHG mobility, reducing the structural defects in epilayer and optimizing device processing of *p*-channel MOSFET are necessary.

In summary, the polarization-induced 2DHG at the lower interface of InGaN/GaN heterostructure was successfully extracted from the optimized structures. The existence of 2DHG was confirmed by theoretical simulation and *C-V* measurement. The *p*-channel InGaN/GaN heterostructure MOSFET based on the 2DHG was first demonstrated by using Al_2_O_3_ as the gate dielectric. The transistor shows a high drain-source current of 0.51 mA/mm and ON/OFF ratio of two orders of magnitude at 300 K. The polarization-induced *p*-channel behaviors are also verified by the well operation for FET at temperature as low as 8 K, which proves the successful realization of 2DHG channel induced by polarization effect. We mention that a theoretical mobility of 2DHG for InGaN/GaN heterojunction is approximately 700 cm^2^/Vs at 66 K, and the theoretical output current of *p*-channel FET can be as high as approximately 100 mA/mm for a gate length of 0.5 μm^25^. The progress of the present FETs based on InGaN is still relatively at the early stage. The current work on the *p*-channel MOSFET by using InGaN/GaN heterojunctions opens a promising route for the development of the nitride-based complementary ICs.

## Methods

### InGaN/GaN heterojunction growth and characterization

The investigated heterojunction was deposited on a 2-μm-thick high-resistance GaN (HR-GaN) template by using the metal organic chemical vapor deposition (MOCVD). Before InGaN deposition, a long-time growth interrupt in both nitrogen and ammonia ambient was introduced to polish the interface. The growth interrupt can improve the interface quality, which was confirmed in our previous study^28^,^29^. Then a super-thin unintentionally doped GaN (UID-GaN) spacer layer with the thickness of 5 nm was grown with an ultra-flat morphology. To compensate the high-density *n*-type background concentration and serve as a source of the holes, slightly Mg-doping is performed for the strained InGaN layer^26^. A 5-nm-thick p-type GaN cap layer was used to screen surface trap effects and enable the formation of ohmic contacts. The material properties were characterized by XRD (Panalytical Xpert PRO XRD system), TEM (JEM-2000EX operated at 200 kV). The TEM sample was prepared by FIB process (Hitachi FB-2100).

### InGaN/GaN p-channel FET fabrication and characterization

The Schottky and MOSFET were fabricated by using the standard semiconductor device process technique. Before processing, the sample was annealed in nitrogen ambient at 700 °C for 15 min for Mg acceptor activation. The device was firstly isolated by the chlorine-based inductively coupled plasma dry etching. Then, Ni/Au (20/30 nm) stacks were deposited by electron-beam (EB) evaporation, followed by annealing at 500 °C for 10 min in air ambient to form ohmic contacts. For the Schottky-type devices, Ti/Au (40/110 nm) bilayers were deposited by EB evaporation as the gate metal stacks. For MOSFET, a 60-nm Al_2_O_3_ was deposited by atomic layer deposition as the gate dielectric. The microscopic image of the resulting device is shown in the inset of Fig. 4 with a gate width *W*_*G*_ = 200 μm, a gate length *L*_*G*_ = 20 μm, a source-gate separation *L*_*SG*_ = 6 μm, and a drain-gate separation *L*_*DG*_ = 6 μm. The *C-V* characteristic for the Schottky contact and MOSFET were measured by Agilent LCR meter (4284A). The DC output performance of the MOSFET was characterized by Keithley 2636A semiconductor parameter analyzer using a three-point probe method at 300 K and 8 K with a cryogenic refrigerator.

## Additional Information

**How to cite this article**: Zhang, K. *et al.* P-Channel InGaN/GaN heterostructure metal-oxide-semiconductor field effect transistor based on polarization-induced two-dimensional hole gas. *Sci. Rep.* **6**, 23683; doi: 10.1038/srep23683 (2016).
